# Socio-demographic and health-related factors associated with cognitive impairment in the elderly in Taiwan

**DOI:** 10.1186/1471-2458-11-22

**Published:** 2011-01-11

**Authors:** Ming-Shiang Wu, Tsuo-Hung Lan, Chun-Min Chen, Herng-Chia Chiu, Tzuo-Yun Lan

**Affiliations:** 1 Institute of Population Health Sciences, National Health Research Institutes, 35 Keyan Road, Zhunan Town, Miaoli County 350, Taiwan; 2 School of Medicine, National Yang-Ming University, No.155, Sec.2, Linong Street, Taipei 112 Taiwan; 3 Graduate Institute of Healthcare Administration, Kaohsiung Medical University, Kaohsiung, 100, Shih-Chuan lst Road, Kaohsiung 807, Taiwan

## Abstract

**Background:**

Cognitive impairment is an age-related condition as the rate of cognitive decline rapidly increases with aging. It is especially important to better understand factors involving in cognitive decline for the countries where the older population is growing rapidly. The aim of this study was to examine the association between socio-demographic and health-related factors and cognitive impairment in the elderly in Taiwan.

**Methods:**

We analysed data from 2119 persons aged 65 years and over who participated in the 2005 National Health Interview Survey. Cognitive impairment was defined as having the score of the Mini Mental State Examination lower than 24. The χ^2 ^test and multiple logistic regression models were used to evaluate the association between cognitive impairment and variables of socio-demography, chronic diseases, geriatric conditions, lifestyle, and dietary factors.

**Results:**

The prevalence of cognitive impairment was 22.2%. Results of multivariate analysis indicated that low education, being single, low social support, lower lipid level, history of stroke, physical inactivity, non-coffee drinking and poor physical function were associated with a higher risk of cognitive impairment.

**Conclusion:**

Most of the characteristics in relation to cognitive impairment identified in our analysis are potentially modifiable. These results suggest that improving lifestyle behaviours such as regular exercise and increased social participation could help prevent or decrease the risk of cognitive impairment. Further investigations using longitudinal data are needed to clarify our findings.

## Background

The older population is rapidly growing throughout the world. By 2020 it is estimated that the elderly will exceed 1 billion, and 700 million of them will be in the developing countries [1]. As for other developing or newly developed countries, Taiwan is facing the problem of population aging. The proportion of people aged 65 years and over in Taiwan reached 7% in 1993, the WHO benchmark of an aged nation. Currently the percentage of older population has increased to 11% and is projected to hit 14% by 2018 [2].

The increasing number of the older people has imposed a huge health impact on the society, as longer survival is also accompanied by the greater decline in health and different domains of function [3]. For the cognitive function, evidence from previous studies has indicated that cognitive function begins to decline from the age of 50 and accelerates after age 65 [4]. For example, the prevalence of cognitive impairment in the US is 19.2% in those aged 65 to 74, 27.6% in 75 to 84, and 38% in 85 and over [4], while the prevalence ranges widely between 9.9% to 45.7% in people aged 60 to 108 years in China and Taiwan [5,6]. All these surveys demonstrate a clear increase in cognitive decline with age.

Given the fact that cognitive decline is an age-related condition, many previous studies have focused on the factors associated with this decline. In general, cognitive function is influenced by a variety of factors, mainly female, older age, low education level, and a history of stroke [7,8]. In addition, smoking, alcohol consumption, physical inactivity, depression, impaired physical function, and lack of social support are also in relation to the decline of cognitive function [9-11].

In Taiwan results from some studies of cognitive function have identified several factors. Those who are older, female, have a lower level of education, engage in lower social activities, and have depressive symptoms are more likely to have cognitive impairment [12].

Although a lot of studies have been conducted to better understand the relationship between health and social factors and cognitive function, they only focus on some specific factors and studies with a wide variety of factors have been relatively rare [13-15]. In particular, in Taiwan the majority of research on risk factors for cognitive function has been limited to studies with small sample and local areas. Using data from the nationwide study with a wide range of characteristics potentially associated with cognitive function would greatly help the identification of key factors about cognitive decline. Therefore, we aimed to analyze data from the National Health Interview Survey to evaluate the association between socio-demographic and health-related factors and cognitive function in the older population in Taiwan.

## Methods

### Study sample

Data used in the study was from the National Health Interview Survey conducted in 2005 in Taiwan. The study sample was selected using a multi-stage stratified systematic sampling design, providing it to be nationally representative [16]. All family members of the selected household were personally interviewed by well-trained interviewers. The whole data has been released for public use and can be obtained through http://nhis.nhri.org.tw. A total of 24726 persons completed the survey with a response rate of 80.6%. All study participants signed the informed consent. This study analyzed 2727 study participants aged 65 years and over [17].

### Measure of cognitive function

The Mini-Mental State Examination (MMSE) was used to assess cognitive status. MMSE includes 11 items that give a total score ranging from 0 to 30 points, with the higher scores representing better cognitive function [18]. Of the 2727 study participants, 232 were excluded because of proxies. Because the MMSE is performance-based and needs to be administered only to study participants themselves, the use of a proxy is not allowed [18,19]. The main reasons for those being unable to answer questions were: the presence of serious illness or physical frailty (64 persons); inability to communicate due to serious mental or psychological impediments (57 persons); inability to communicate due to severe hearing problems (81 persons); and other miscellaneous reasons (30 persons). In addition, 376 who answered too few questions to accurately estimate their cognitive function were also excluded, leaving 2119 (77.7%) in our analysis. Of the 2119 study participants, 973 answered full questions for all 30 potential points (45.9%), and 1145 answered questions for 27-29 potential points (54.1%). The most common reason for the incompletion was illiteracy (69.2%). To consider the potential effect from both the incompletion and educational difference, we made an adjustment similar to that used in a previous study [12]. The lower cut-off points of cognitive impairment were set separately for those with incompletion and those with incompletion and illiteracy compared to those answering all questions. For example, the cut-off point was 23 instead of 24 for those who answered 29 potential points, and 21 for those who also answered 29 potential points but were illiterate.

### Other measures

Other measures collected in the study were socio-demography, health status, lifestyle factors, diet, and body mass index (BMI). These measures were categorized at analysis as binary, tertiled, or quartiled variables based on their frequency distributions. Socio-demography includes age, gender, education level, marital status, and social support. Age was dichotomised as 65-74 years and 75 years and over. Education level was divided into illiterate, elementary school, and high school and above. Marital status was dichotomized as single and married. Social support was measured using questions from the social resource section of the OARS (Older American Resources and Service Centre) Multidimensional Functional Assessment Questionnaire (MFAQ) [20]. Eight items regarding the frequency of meetings with the relatives including children, siblings, and others, social contact with neighbours and friends, and attendance at clubs, other organizations, and church were asked, with never coded as 0, rarely or sometimes as 1, and often or regularly as 2. The eight responses were then used to create a summary score of social support ranging from 0, meaning not able to receive any support, to 16, meaning able to receive all supports. At analysis, social support scores were further grouped into low (0-5), moderate (6-8), and high (9-16).

The information of health status includes chronic diseases, physical function, mental health, and self-rated health. Chronic diseases were eleven diseases diagnosed by the physician including hypertension, diabetes, stroke, heart disease, hyperlipidemia, renal disease, gout, chronic respiratory disease, osteoporosis, cancer, and cataract. Similar to those employed in the US NHIS, 22 questions about the level of difficulty in performing six Activities of Daily Living (ADL), eight Instrumental Activities of Daily Living (IADL), and eight mobility tasks were used to measure physical function. At analysis, physical function was further categorized as normal (without any difficulty); mild disability (with difficulty in at least one mobility task); moderate disability (with difficulty in both IADL and mobility tasks), and severe disability (with difficulty in ADL, IADL, and mobility tasks). Mental health was evaluated using the Centre For Epidemiologic Studies Depression Scale (CES-D) [21]. We used the 10 itemed CESD [22]. which has been tested with good validity and reliability [23]. Each item is rated from 0 to 3 and the total score ranges from 0 to 30, with higher scores representing more severe depression. Study participants with scores ranging from 0 to 9 were defined as 'no depression', while those with 10 to 30 were 'depression' [23]. Self-rated health was grouped from five response categories into three categories: poor, fair, and good (including good, very good, and excellent).

Lifestyle factors include alcohol consumption, cigarette smoking, betel quid chewing, leisure-time physical activity, and occupational physical activity. Alcohol consumption was defined as the estimated intensity and was calculated as the frequency of alcohol consumption per month (2 for once or twice every month, 4 for once weekly, 13 for drink once every 2 to 3 days, and 30 for drinking every day) multiplied by the degree of alcohol drinking (1 for shallow sip but not drunk, 2 for half-drunk, and 3 for often drunk). Based on the estimated intensity, all participants were classified as non-drinkers (0), light drinkers (2-13), and moderate to heavy drinkers (26-90). Cigarette smoking was measured using the consumed packs of cigarette per year. Study participants were grouped as never smokers, ex-smokers, light smokers (< 274 packs per year), and heavy smokers (> 275 packs per year). Betel quid chewing was categorized as never chewers and ever chewers. Leisure-time and occupational physical activity was measured using total energy expended on all leisure-time or occupational activities in a week. The Metabolic equivalent (MET)[24] was used to estimate the energy expended by each activity. The leisure-time physical activity was categorized as no, low (< 915 calories per week), and moderate to vigorous amount (> 914 calories per week). Similarly, occupational physical activity was classified as no, low (< 549 calories per week), moderate (550 - 2939 calories per week), and vigorous amount (> 2940 calories per week).

Dietary measures include weekly consumption of milk, fruits and vegetables, coffee, tea, vitamin, and calcium supplements, and BMI. The intakes of milk, vitamin, and calcium supplements were dichotomized as yes or no. Based on the frequency of weekly consumption, the consumption of both fruits and vegetables was divided into low (two times or less in fruits and five times or less in vegetables in a week), medium, and high frequencies (a daily basis for both fruits and vegetables). Similarly, the intakes of coffee and tea were categorized, based on the frequency of consumption in a week, as less than and more than one time. BMI was calculated as weight in kilograms divided by square of height in meters and were categorized as low (< 19), normal (19 to 24), and overweight or obese (> 24), according to the criteria set by the Department of Health in Taiwan [25].

### Statistical analysis

The χ^2 ^test was used to describe the unadjusted associations between all variables and cognitive function. The variables that were demonstrated to be associated with cognition in the univariate analyses were included in the multivariate logistic regression models.

Although we had excluded 376 who only answered few questions of the MMSE, the scores for some of the remaining 2119 who did not complete all questions because of illiteracy or other reasons needed further adjustments. Similar to the adjustments made in a previous study [12]. Incompletion of the questions and illiteracy were considered in the cut-off point of cognitive impairment for those failing to complete all questions and/or being uneducated. For example, the cut-off point was set at 23 instead of 24 for those who answered 29 potential points and were literate, whereas the cut-off point was set at 21 for those who also answered 29 potential points but were illiterate. All statistical analyses were carried out using SPSS 15.0 statistical software. Statistical significances were considered at the < 0.05 level.

## Results

The mean scores of MMSE were 26.9 (standard deviation = 2.5) for normal cognition and 18.0 (standard deviation = 3.8) for cognitive impairment, respectively. There were more men than women in our study sample and the mean age of participants was 73.3 years (standard deviation = 5.9). The prevalence of cognitive impairment was 22.2%. The results of univariate analyses between all variables and cognitive impairment are shown in Table 1. Cognitive impairment rates increased with older age and decreased with higher level of education. Women and single persons were more likely to have cognitive impairment as were those with low social support.

**Table 1:** Basic analysis of cognitive impairment and related factors. Data collected in Taiwan, 2005

	n(%)		Cognitive impairment				p-value
			No		Yes		
Age (years)							
65-74	1347	(63.6)	1102	(81.8)	245	(18.2)	0.000
75+	772	(36.4)	545	(70.6)	227	(29.4)	
Gender							
Male	1102	(52.0)	931	(84.5)	171	(15.5)	0.000
Female	1017	(48.0)	716	(70.4)	301	(29.6)	
Educational level							
Illiterate	624	(29.4)	377	(60.4)	247	(39.6)	0.000
Elementary school	997	(47.1)	813	(81.5)	184	(18.5)	
High school and higher	498	(23.5)	457	(91.8)	41	(8.2)	
Marital status							
Single	690	(32.6)	473	(68.6)	217	(31.4)	0.000
Married	1429	(67.4)	1174	(82.2)	255	(17.8)	
Social support							
Low	453	(21.4)	309	(68.2)	144	(31.8)	0.000
Moderate	952	(44.9)	735	(77.2)	217	(22.8)	
High	714	(33.7)	603	(84.5)	111	(15.5)	
Hypertension							
No	1216	(57.4)	943	(77.5)	273	(22.5)	0.833
Yes	903	(42.6)	704	(78.0)	199	(22.0)	
Diabetes							
No	1778	(83.9)	1394	(78.4)	384	(21.6)	0.089
Yes	341	(16.1)	253	(74.2)	88	(25.8)	
Heart disease							
No	1744	(82.3)	1363	(78.2)	381	(21.8)	0.306
Yes	375	(17.7)	284	(75.7)	91	(24.3)	
Hyperlipidemia							
No	1654	(78.1)	1258	(76.1)	396	(23.9)	0.000
Yes	465	(21.9)	389	(83.7)	76	(16.3)	
Stroke							
No	2011	(94.9)	1581	(78.6)	430	(21.4)	0.000
Yes	108	(5.1)	66	(61.1)	42	(38.9)	
Physical function							
Normal	958	(45.2)	841	(87.8)	117	(12.2)	0.000
Mild limitation	468	(22.1)	381	(81.4)	87	(18.6)	
Moderate limitation	523	(24.7)	337	(64.4)	186	(35.6)	
Severe limitation	170	(8.0)	88	(51.8)	82	(48.2)	
Depressive symptoms							
No	1732	(81.7)	1399	(80.8)	333	(19.2)	0.000
Yes	387	(18.3)	248	(64.1)	139	(35.9)	
Self-rated health							
Poor	468	(22.1)	311	(66.5)	157	(33.5)	0.000
Fair	876	(41.3)	692	(79.0)	184	(21.0)	
Good	775	(36.6)	644	(83.1)	131	(16.9)	
Alcohol consumption							
Non-drinkers	1803	(85.1)	1390	(77.1)	413	(22.9)	0.067
Light drinkers	166	(7.8)	141	(84.9)	25	(15.1)	
Moderate to heavy drinkers	150	(7.1)	116	(77.3)	34	(22.7)	
Cigarette smoking							
Never smokers	1512	(71.4)	1144	(75.7)	368	(24.3)	0.003
Ex-smokers	231	(10.9)	189	(81.8)	42	(18.2)	
Light smokers	196	(9.2)	160	(81.6)	36	(18.4)	
Heavy smokers	180	(8.5)	154	(85.6)	26	(14.4)	
Betel quid chewing							
Never chewers	1935	(91.3)	1509	(78.0)	426	(22.0)	0.200
Ever chewers	184	(8.7)	138	(75.0)	46	(25.0)	
Leisure-time physical activity							
No	846	(39.9)	598	(70.7)	248	(29.3)	0.000
Low amount	614	(29.0)	486	(79.2)	128	(20.8)	
Moderate to vigorous amount	659	(31.1)	563	(85.4)	96	(14.6)	
Occupational physical activity							
No	1694	(79.9)	1311	(77.4)	383	(22.6)	0.162
Low amount	146	(6.9)	124	(84.9)	22	(15.1)	
Moderate amount	139	(6.6)	107	(77.0)	32	(23.0)	
Vigorous amount	140	(6.6)	105	(75.0)	35	(25.0)	
Milk consumption							
No	600	(28.3)	454	(75.7)	146	(24.3)	0.085
Yes	1519	(73.7)	1193	(78.5)	326	(21.5)	
Fruits and vegetables consumption							
Low frequency	106	(5.0)	69	(65.1)	37	(34.9)	0.000
Medium frequency	792	(37.4)	580	(73.2)	212	(26.8)	
High frequency	1221	(57.6)	998	(81.7)	223	(18.3)	
Coffee intake							
No	1415	(66.8)	1034	(73.1)	381	(26.9)	0.000
Less than once per week	439	(20.7)	370	(84.3)	69	(15.7)	
More than once per week	265	(12.5)	243	(91.7)	22	(8.3)	
Tea intake							
No	770	(36.3)	548	(71.2)	222	(28.8)	0.000
Less than once per week	373	(17.6)	284	(76.1)	89	(23.9)	
More than once per week	976	(46.1)	815	(83.5)	161	(16.5)	
Multivitamin intake							
No	1665	(78.6)	1263	(75.9)	402	(24.1)	0.000
Yes	454	(21.4)	384	(84.6)	70	(15.4)	
BMI (kg/m^2^)							
Low (< 19)	113	(5.3)	74	(65.5)	39	(34.5)	0.004
Normal (19 to 24)	965	(45.5)	748	(77.5)	217	(22.5)	
Overweight or obese (> 24)	1041	(49.1)	825	(79.3)	216	(20.7)	

A history of stroke and lower lipid level were also associated with cognitive impairment (p < 0.001). A clear gradient relationship was observed between the decline of physical function and impaired cognitive function (p < 0.001). Those with depressive symptoms and poorer self-rated health also had higher prevalence of cognitive impairment (p < 0.001). Non-smokers tended to have higher rates of cognitive impairment than smokers (p = 0.003), whereas exercisers had lower proportion of impaired cognitive than sedentary individuals (p < 0.001). There was a reverse relationship between cognitive impairment and the intakes of vegetables and fruits, coffee, and tea (p < 0.001). Individuals taking multivitamin and having higher BMI had lower percentages of having cognitive problems (p < 0.004).

Table 2 presents the results of multiple logistic regression analysis. Among the 17 variables with statistical significance in the univariate analysis, eight were independently associated with cognitive problems. In the final model, those who were older and single, had lower level of education, lower social support, and lower lipid level, and were with a history of stroke, impaired physical function, physical inactivity, and no coffee intake were at a significantly higher risk of cognitive impairment.

**Table 2:** Multiple logistic regression of cognitive impairment and related factors. Data collected in Taiwan, 2005

	Cognitive impairment			
	
	**OR**^**b**^	**95%CI**^**b**^		p
			
		Lower	Upper	
Age(years)(65-74 ^a^)				
75+	1.27	0.98	1.63	0.068
Gender (Male ^a^)				
Female	1.16	0.84	1.59	0.375
Educational level (Illiterate ^a^)				
Elementary school	0.51	0.39	0.67	0.000
High school and higher	0.25	0.16	0.38	0.000
Marital Status (Single ^a^)				
Married	0.76	0.60	0.98	0.032
Social support (Low ^a^)				
Moderate	0.87	0.65	1.16	0.352
High	0.62	0.45	0.87	0.005
Hyperlipidemia (No ^a^)				
Yes	0.65	0.48	0.88	0.005
Stroke (No ^a^)				
Yes	1.69	1.05	2.72	0.031
Physical function (Normal ^a^)				
Mild	1.34	0.96	1.86	0.081
Moderate	2.32	1.70	3.16	0.000
Severe	3.10	2.00	4.80	0.000
Depressive symptoms (No ^a^)				
Yes	1.34	1.00	1.79	0.054
Self-rated health (Good ^a^)				
Fair	0.95	0.72	1.25	0.697
Poor	1.09	0.78	1.53	0.611
Cigarette smoking (Never ^a^)				
Ex-smokers	1.09	0.70	1.70	0.690
Light smokers	1.05	0.66	1.64	0.848
Heavy smokers	0.75	0.45	1.25	0.265
Leisure-time physical activity (No ^a^)				
Low amount	0.73	0.56	0.96	0.026
Moderate to vigorous amount	0.72	0.53	0.97	0.029
Fruits and vegetables consumption (Low frequency ^a^)				
Medium frequency	0.84	0.51	1.37	0.481
High frequency	0.81	0.49	1.33	0.403
Coffee intake (No ^a^)				
Less than once per week	0.68	0.49	0.94	0.018
More than once per week	0.51	0.31	0.83	0.007
Tea intake (No ^a^)				
Less than once per week	1.14	0.82	1.59	0.437
More than once per week	0.99	0.75	1.30	0.940
Multivitamin intake (No^a^)				
Yes	0.82	0.59	1.13	0.226
BMI (kg/m^2^) (Low^a^)				
Normal (19 to 24)	0.81	0.51	1.28	0.365
Overweight or obese (> 24)	0.66	0.41	1.06	0.083

^a ^Reference group.

^b ^Adjusted odds ratios and 95% confidence interval.

## Discussion

This study identified several characteristics of cognitive impairment. Education level, marital status, and social support in the demography, stroke, blood lipids, and physical function in the health status, physical activity in the lifestyle, and coffee consumption in the diet were all associated with cognitive impairment.

Our study found that those with low education were more likely to have cognitive problems than their counterparts. Results from previous studies have also shown that low education is associated with increased risk of cognitive impairment [7,8,12]. The relationship between education level and cognitive function could be due to lifestyle factors. People with higher education might adopt healthier lifestyles that are associated with good cognitive function [8]. However, our finding showed that education remained significantly associated with cognitive function after controlling for lifestyle factors. The possible explanation is that lower education could lead to lower memory capacity. People with higher education levels could readily maintain or increase cognitive functional development as their brains are frequently stimulated to think during the daily lives [26,27].

Evidence from studies of cognitive function and marital status indicates that, for both gender, married people are less likely to suffer from dementia than those who are divorced, separated, or single [28]. Consistent with previous finding, our results also revealed that married persons were less likely to have cognitive impairment than single persons. There have been some explanations proposed for the association. Married individuals are at lower risk of developing cognitive problems mainly because of better mental conditions and lifestyle behaviours [29,30]. However, after controlling for mental and lifestyle factors in our study we still found that marital status was significantly associated with cognitive impairment. It is possible that sharing one's life with a partner results in stimulating brain activities and the growth of neurons. As a result, married persons could have lower speed of cognitive decline.

This study also confirmed evidence from previous studies [31,32] that persons with low social support have a higher risk of cognitive impairment. It is thought that maintenance of social ties and active participation in community activities is associated with higher memory ability and prevention of cognitive decline [33]. It is also possible that the maintenance of good social relations with friends and relatives in old age results in continued mental stimulation and an increase in cerebral neuronal growth that delays cognitive decline [31].

There has been clear evidence that age and gender are independent risk factors of cognitive function [7,8]. However, these two factors were not significantly associated with cognitive impairment in our study. In our multivariate logistic regression, older age only had a borderline significant association with cognitive impairment. The possible reason is that age was in relation to education level (Data not shown). In Taiwan, educational attainment was improved after the Second World War and thus higher level of education are associated with younger age groups. The effect of age was therefore partly explained by education. Similarly, there was a significant association between education level and gender in which women had lower educational attainment than men. In addition, gender was also found to be associated with physical functioning in which men had better physical function than women. As a result, once age, gender, education level, and physical function were all considered in the model, gender became statistically non-significant.

Consistent with previous studies, our study found that stroke is associated with cognitive impairment in the older persons. Stroke sufferers have relatively higher risk to develop cognitive decline [34], including cognitive impairment and dementia. The possible reason is that stroke could damage the brain vessels which affect functions including memory and finally lead to cognitive problems and dementia [35,36].

In contrast to the above findings, the relationship between blood lipid level and cognitive function was different from that in previous studies in which hyperlipidemia increases the risk of cognitive impairment [37,38]. To better understand the reason that hyperlipidemia was observed as a protecting factor against cognitive impairment in our study, we examined the associations between hyperlipidemia and all other variables. We found a strong gradient relationship between BMI and hyperlipidemia, with low BMI having low likelihood of hyperlipidemia (Data not shown). Since the effect of BMI was explained by the level of blood lipids, no association of BMI and cognitive impairment was observed in our study. Previous research has demonstrated a relationship between low BMI and the decline in cognitive function [39,40]. Low BMI could result in cerebral damages that lead to reduced cognitive function [39]. Therefore our finding of the link between lower lipid level and cognitive impairment could be mediated by BMI. In addition to the effect from BMI, the association between hyperlipidemia and cognitive function may be biased by excluding those without or with incomplete cognitive data in our study. Our result could also be explained by the use of medicine for the treatment of hyperlipidemia. Results from some previous studies have indicated that relatively better cognition found in patients with hyperlipidemia may be associated with the use of statin [41,42]. Obviously, further investigations focusing on the association between hyperlipidemia and cognitive function are still needed.

It has been widely accepted that the decline of physical function increase the risk of cognitive decline [43]. Similar results were also observed in our study. The possible speculation is that both physical and cognitive functions affect each other. Cognitive function is necessary for the performance of certain daily activities. On the other hand, the performance of these activities can maintain or improve cognitive ability [44].

Many population-based studies have demonstrated a beneficial effect of exercise on the delaying of cognitive decline [11,45]. Our data also revealed that great amount of weekly leisure-time physical activity was associated with cognitive impairment. There have been some speculations about this association. Exercise can improve cognitive function by raising cardiovascular function which enhances brain processing speed, memory, mental flexibility, and cognitive function [45]. Exercise can also increase cerebral supply capacity and improve cell proliferation [46]. It is also possible that increased physical activity can increase the activities of frontal and prefrontal areas in the brain, which are responsible for planning, co-ordination, control memory, and communications, and further improve the cognitive function [36].

We found that coffee drinker was associated with a decreased risk of cognitive impairment. Recent research has also demonstrated the protective effect of coffee on cognitive decline [47,48]. The possible speculation is that coffee stimulates the brain's cholinergic system to control the β-amyloid-induced damage to brain cells, thereby helping to maintain the memory function [49]. Coffee can also stimulate the cerebral nervous system and has a positive effect on vigilance, attention, mood and arousal [50]. Since coffee consumption in the older population in Taiwan is not as popular as that in the western countries, the gradient relationship between coffee consumption and the cognitive decline found in many studies is not examinable in our study. However, we still found that older persons who drank coffee one or more times per week had a lower risk of cognitive impairment than non-coffee drinkers.

### Study strengths and limitations

Current data about cognitive function in non-western countries is extremely limited [51-53]. Our study makes an important contribution to these countries by using a nationally representative dataset to investigate factors associated with cognitive function in older adults. Furthermore, in Taiwan research investigating risk factors associated with cognitive function is sparse. Only one similar study was conducted in southern Taiwan [12]. Its study sample was limited to local older people aged between 65 and 74. All these add the credibility of our findings.

On the other hand, data analyzed in this study came from a cross-sectional survey and, as a result, it is not able to make the causal inferences from our associations. A second issue is the possible effect of non-participation in the cognitive test on our results. This exclusion of some sample inevitably raises the possibility of selection bias due to missing data. Given the fact that MMSE is limited to people who are measurable, it is possible that factors for people with severe cognitive impairment are not the same as those identified in our study for people with mild to moderate cognitive impairments. Therefore, our results with factors of cognitive impairment may not be generalizable to people with severe cognitive impairment and should be interpreted with caution.

## Conclusions

An important merit of this study was to demonstrate the independent association of a wide range of sociodemographic and health-related factors on cognitive function in the older persons in Taiwan. Most of the characteristics identified in our study are modifiable including factors of social support, some health condition, lifestyle, and diet. Hence, the factors may be further adopted to evaluate the risk of cognitive impairment. Further longitudinal studies are needed to investigate the causal relationships between these factors and cognitive function.

## Competing interests

The authors declare that they have no competing interests.

The 2005 NHIS Taiwan was funded by the National Health Research Institutes, Taiwan, the Bureau of Health Promotion, Department of Health, Taiwan, and the Food and Drug administration, Department of Health, Taiwan.

## Authors' contributions

T.-Y. L. was an investigator of the NHIS study in Taiwan. He participated in designing the study, collected and analysed data, wrote the first draft of the paper, and led the critical review and revision of the paper. M.-S. W. was the research assistant of the study. He contributed to the design of study, collected and analysed data, and contributed to the drafting and critical reviewing of the paper. The remaining authors (T.-H. L., C.-M. C., and H.-C. C.) made substantial contributions to the conception and design, interpretation of data, and critically revised the intellectual content of the manuscript. All authors have approved the final version of the manuscript.

## Pre-publication history

The pre-publication history for this paper can be accessed here:

http://www.biomedcentral.com/1471-2458/11/22/prepub
